# Use of nutraceuticals in the stallion: Effects on semen quality and preservation

**DOI:** 10.1111/rda.13934

**Published:** 2021-04-05

**Authors:** Marilena Bazzano, Fulvio Laus, Andrea Spaterna, Andrea Marchegiani

**Affiliations:** ^1^ School of Biosciences and Veterinary Medicine University of Camerino Matelica MC Italy

**Keywords:** cooled semen, equine semen, frozen semen, nutraceutical, stallion

## Abstract

Nutritional supplements are widely used in the equine industry with the aim of improving horse health, sports or reproductive performances. Over the years, a number of studies have focused on investigating the effects of several dietary compounds on the quality and preservation of stallion semen. This paper reviews the literature available on the use of nutritional supplementation for the improvement of reproductive performance and semen quality in equine species, critically appraising the benefits and negative effects of several compounds found in complementary feeds such as PUFAs from different sources, vitamins and antioxidants, carnitine and botanical extracts. Different nutraceuticals have been highlighted to improve stallion fertility by providing optimal levels of antioxidants, with the most promising results obtained by the combination of PUFAs and antioxidants that resulted to be essential for the maintenance of normal reproductive functions and the reduction of cryodamage in cooled and frozen equine semen.

## 1 INTRODUCTION

Over the past decades, progress in stallion semen preservation has contributed to the success of artificial insemination (AI) in equine reproduction (Aurich, 2012).

The development of new AI techniques alongside with relevant advances made in cryoprotection of cooled and frozen semen have increased the quality of stallion semen and improved the reproductive efficiency of horses (Alvarenga et al., 2016; Aurich, 2005, 2008). Cooling and freezing storage of semen is not immune from deleterious effects on spermatic cells such as a decrease in cell viability, motility and fertility (Aurich, 2005).

In fact, cryopreservation may increase oxidative damage and lipid peroxidation of cell membranes causing a decrease in spermatozoa lifetime and fertility, with negative reflections on artificial insemination pregnancy rates (Peña et al., 2019). To cope with this, different techniques have been developed over time to provide new cryopreservation protocols and methods to maintain suitable fertility (Alvarenga et al., 2016).

Apart from semen extenders and freezing protocols, in recent years a number of nutritional supplements have been used in attempt to improve stallion fertility by optimizing the use of nutrients in different metabolic pathways (de Arruda et al., 2010; Freitas, Bouéres, et al., 2016; Freitas, Pignataro, et al., 2016).

The North American Veterinary Nutraceutical Council defined nutraceuticals in veterinary medicine as ‘substances produced in a purified or extracted form and administered orally to patients to provide agents for normal body structure and function and administered with the intent of improving the health and well‐being of animals’. Recently, nutraceuticals have become remarkably popular in both human and veterinary medicine and substances of vegetable origin, minerals and vitamins are available on the market for the management of male infertility (Freitas & De Oliveira, 2018; Ko & Sabanegh, 2014).

Dietary intervention can play a critical role in replacing nutritional deficiencies that may affect sperm production and function, as well as antioxidant capacity (Trillaud‐Geyl et al., 2015). There is a growing evidence that nutraceuticals interact with reproductive function in males and different authors have explored nutraceutical and dietary interventions with regards to semen preservation. The aim of this paper is to provide an updated review on nutraceuticals used in attempt to improve reproductive performance and quality of cooled and frozen semen of breeding stallions.

### 1.1 PUFAs

Equine semen lipid composition was found to include high levels of polyunsaturated fatty acids (PUFAs), in particular docosahexaenoic acid (DHA, omega‐3) and docosapentaenoic acid (DPA, omega‐6) (Komarek et al., 1965; Wolkmer et al., 2019). Horses, as other animals, are unable to self‐synthesize PUFAs and have to rely on PUFAs precursor in their diet (Trillaud‐Geyl et al., 2015).

The optimal concentrations of omega‐3 as well as DHA‐to‐DPA ratio in horse diets have still to be elucidated (Trillaud‐Geyl et al., 2015). Forages represents the main constituent of horse aliment and are characterized by an omega‐6 to omega‐3 ratio ranging from 1.3:1.0 to 1.0:6.3, depending on forage composition, time of harvesting and characteristics of the growing season (Clapham et al., 2005; Dewhurst et al., 2001; Duckett et al., 2013; Elgersma et al., 2003).

Regarding specific omega‐3 activity on spermatic cells, DHA maintains flexibility, compressibility, deformability and elasticity of spermatozoa (Parks & Graham, 1992), and, together with DPA, it is responsible for membrane stability during cooling and cryopreservation process (Parks & Lynch, 1992). On the basis of these properties, a number of studies aimed at investigating the effect of PUFA‐enriched supplementation on the quality of semen.

Brinsko and collaborators (Brinsko et al., 2005) evaluated the addition of 250 g DHA‐enriched nutraceutical to everyday diet in a 14‐week feeding cross‐over trial. Eight fertile breeding stallions were randomly assigned to one of two treatment groups (supplemented and control, respectively). After 14 weeks washout, the groups were reversed for an additional 14‐week feeding trial. The study showed a statistically significant increase in semen DHA levels and a higher DHA‐DPA ratio in those stallions fed with DHA‐enriched nutraceutical. However, the overall quality of fresh semen was not affected by the supplementation, and total and progressive motility did not differ between supplemented and control stallions after 24 hr of cooling storage. Nevertheless, DHA was responsible for a marked, though not statistically significant, improvement in spermatozoa velocity and progressive motility in supplemented stallions after 48 hr of cooling. Moreover, in those stallions having <40% progressive sperm motility after 24 hr of cooling before starting supplementation, a significant improvement in progressive sperm motility was observed both after 24 and 48 hr of cooling storage. Interestingly, the authors found that nutraceutical also seemed to improve frozen‐thawed semen motility, thus stating that DHA supplementation could exert beneficial effects on semen quality for those stallions whose sperm do not tolerate cryopreservation (Brinsko et al., 2005).

Other authors studied the influence of dietary PUFAs supplementation in horses, obtaining controversial results.

Rodrigues and collaborators (Rodrigues et al., 2017) assessed the effects of linseed oil supplementation, as a source of DHA, on the quality of fresh, cooled and frozen‐thawed semen from ten Mangalarga Marchador stallions. In this cross‐over study, horses from the experimental group received 150 ml of linseed‐based oil supplement to their diet for 60 days and, after a sixty‐day washout period, groups were reversed. The study found no effect of PUFA supplementation on the quality of cooled semen. According to Rodrigues and collaborators, thiobarbituric acid reactive substances (TBARs) were significantly increased over time in stallion fed with this nutraceutical. Furthermore, motility, vigour, viability, acrosome integrity and osmotic tolerance were found to be higher in semen from supplemented horses in comparison with control group. To explain this finding, authors hypothesized a major sperm susceptibility to ROS as a consequence of the higher levels of PUFAs within cell membranes.

Schmid‐Lausigk and Aurich evaluated the ability of PUFAs supplementation to preserve semen quality during the winter, as this season is known to alter the composition of sperm lipid membrane in stallions (Mislei et al., 2020; Schmid‐Lausigk & Aurich, 2014). Starting from November, the diet of six stallions was enriched for 84 days with 100 ml linseed oil once a day while five stallions served as controls. According to Authors' findings, the quality of frozen‐thawed semen decreased from November to February irrespective of supplementation, whereas sperm motility and membrane integrity were preserved at cooling temperature in supplemented horses during early winter.

Apart from DHA‐to‐DPA ratio, another factor that deserves to be considered when paralleling results of similar experiments is the source of the omega‐3 oil.

Grady and collaborators (Grady et al., 2009) obtained controversial results evaluating different sources of dietary DHA in horses. In the study, horse diets were enriched with either algae‐ plus flaxseed‐based or fish‐based dietary supplement, but no overall significant improvement was observed in motility, integrity of membrane and morphology of spermatozoa, irrespective of either supplementation or DHA source. The authors ascribed these results to a higher susceptibility of spermatozoa to lipid peroxidation as a consequence of the increased PUFAs content which predispose to a higher risk of oxidative damage (Grady et al., 2009).

As a different source of DHA, a yeast‐based supplement containing 15 g DHA from a heterotrophically grown microalgae has been administered to stallions in addition to 1,000 IU of Vitamin E plus 2 mg selenium with the aim of assessing possible enhancement in the quality of fresh, cooled, and frozen‐thawed semen (Goedde, 2016). The study, conducted during the summer season, showed that dietary supplementation of DHA, Vitamin E and selenium improved significantly spermatozoa total and progressive motility in both fresh and cooled semen while no significant effect was detected in frozen‐thawed semen (Goedde, 2016).

Pomegranate seed oil (Nouri et al., 2018) has been supplemented as an alternative source of PUFAs in a cross‐over trial. In this study, Nouri and collaborators assessed the quality of fresh, cooled and frozen semen of eight Arabian stallions receiving 200 ml of pomegranate seed oil for ninety days. According to their results, the dietary supplement had no effect on fresh semen but improved spermatozoa membrane integrity and viability in cooled semen compared to controls. Furthermore, post‐thawed semen from supplemented stallions showed a statistically significant improvement of total motility and acrosome status. In the same study, 126 Arabian mares have been inseminated with fresh semen from supplemented (n.61 mares) and control (n.65 mares) stallions. The authors observed an overall first‐cycle pregnancy rate of 64.32%, and found no difference between groups.

Fish oil supplementation has been also preliminary evaluated in Caspian miniature horses. (Paper & Branch, 2014) Stallions receiving this source of PUFAs showed a significant improvement of spermatozoa motility and viability, acrosomal conformation, and plasma membrane integrity. In another study (Kheradmand Garmsir et al., 2014), oral supplementation of fish oil and thyme resulted in an improved quality, increased motility, and improved plasma membrane integrity, of cooled semen of miniature Caspian stallions.

## 2 CARNITINE

L‐carnitine is crucial nutrient for mitochondrial β‐oxidation (Foster & Harris, 1989). The biosynthetic capacity of producing carnitine from lysine and methionine may be a limiting factor in horses and therefore carnitine is often regarded as a conditionally essential nutrient (Trillaud‐Geyl et al., 2015). In stallions, L‐carnitine has been found in epididymal secretions, constituting a reserve of carnitine for equine sperm (Kareskoski & Katila, 2008; Magistrini et al., 1995). In men, carnitine active transportation from the blood to epididymal lumen is regulated by androgens (Enomoto et al., 2002; Jeulin & Lewin, 1996) and the same mechanism is thought to occur in horses. In humans, the beneficial effects of carnitine on the reproductive system are well known (Mongioi et al., 2016). Researchers demonstrated a positive correlation between L‐carnitine, acetyl carnitine and spermatozoa concentration as well as acetyl carnitine and total motile spermatozoa, suggesting L‐carnitine and its derivatives as possible markers of semen quality in horses (Stradaioli et al., 2000). Since seminal carnitine concentration is a direct reflection of dietary carnitine, the same authors investigated the effect of supplementing 20mg of L‐carnitine to horse diet for 60 days. According to semen quality, eight stallions were equally distributed in high motility (HM, more than 50% of progressive motile spermatozoa, and more than 80 × 10^6^ spermatozoa/ml) and less motility (LM, <50% progressive motile spermatozoa, and <80 × 10^6^ spermatozoa/ml) breeder. Carnitine supplementation was responsible for an increase in progressive motile spermatozoa and free seminal plasma carnitine concentration, which was registered only in the LM group. Raw semen and seminal plasma carnitine and acetyl carnitine levels were found to be positively correlated with both sperm concentration and progressive motility; additionally, acetyl carnitine content was positively correlated with total motile morphologically normal spermatozoa. According to these results, oral administration of L‐carnitine could improve semen quality in stallions with poor fertility, although no effect has been observed on fertile stallions (Stradaioli et al., 2004).

## 3 ANTIOXIDANTS AND VITAMINS

Alteration of redox homeostasis has been acknowledged as a substantial trigger of infertility in both man and stallions (Peña et al., 2019). Spermatozoa normally produce reactive oxygen species (ROS) as a by‐product of mitochondrial activity (Tosic & Walton, 1946). A discrepancy in the generation and inactivation balance of ROS have been shown to exert severe detrimental effects on sperm (Baumber et al., 2000). In fact, stallion sperm is characterized by a remarkably powerful mitochondrial activity in comparison with other mammals that predisposes mitochondria to dysfunction and oxidative damage (Davila et al., 2015; Gibb et al., 2014; Swegen et al., 2016), thus negatively affecting sperm function (Peña et al., 2019; Kalyanaraman, 2013).

High levels of ROS in spermatozoa have deleterious consequences on DNA, proteins and lipids, especially phospholipids and cholesterol, causing changes in the permeability and fluidity of membranes (Peña et al., 2019). Lipid peroxidation is widely acknowledged as a major outcome of redox imbalance in aged and cryopreserved spermatozoa (Munoz et al., 2016; Muñoz et al., 2015; Ortega Ferrusola et al., 2009).

Redox balance regulates critical functions of the spermatozoa, such as sperm capacitation, and several studies focused on redox regulation and male fertility (O'Flaherty, 2015). Among antioxidants, the importance of vitamins in spermatogenesis is well‐known; as an example, vitamin E deficiency is responsible for reduced motility and increased number of abnormal spermatozoa. Gee and collaborators assessed the effects of vitamin E supplementation on sperm viability in poor‐freezer stallions. Fifteen stallions were randomly allocated to receive either daily dietary addition of 3,000 UI vitamin E for 14 weeks or standard feed. Based on observed data, supplementation did not produce significant effects on total and progressive motility of raw or frozen‐thawed semen (Gee et al., 2008). The authors found significative improvements in total and progressive motility of 48 hr cooled semen after a warming period of 10–30 min.

Vitamin A and its precursor beta‐carotene are also involved in spermatogenesis and a deficiency can be responsible for reduced mobility and increased abnormal sperm percentage in both stallions and bulls (Ralston et al., 1986). In contrast, no consistent data is available on the effects of vitamin C as unique dietary supplement on stallions' fertility (Ralston et al., ,^1986^, ^1988^).

Studies on dietary supplementation with antioxidant compounds (i.e. selenium and carotene) and vitamins (i.e. vitamin E and vitamin C), eventually blended with PUFAs, revealed improvements in antioxidant defence and reduction in oxidative stress‐related sperm damage in a number of species, including equines (Audet et al., 2004; Eid et al., 2006; Eskenazi et al., 2005; Strzezek et al., 2004; Zubair et al., 2015). On the contrary, other studies found no improvement in semen parameters after dietary antioxidant supplementation for pony and stallions (Contri et al., 2011; Deichsel et al., 2008).

Deichsel and collaborators (Deichsel et al., 2008) investigated the effects of dietary supplementation with antioxidants including tocopherol (300 mg/day), ascorbic acid (300 mg/day), folic acid (12 mg/day) and L‐carnitine (4,000 mg/day) on semen quality in Shetland pony stallions, highlighting no significant change in total spermatozoa count, motility and membrane integrity.

In a study by Contri and collaborators (Contri et al., 2011) ten fertile stallions were divided in two equal groups, one receiving 1,500 mg of alpha‐tocopherol acetate, 360 mg of zinc, and 2.5 mg of organic selenium dietary supplementation for 60 days, and another one receiving no supplement. The raw semen collected from the supplemented group showed a statistically significant improvement in sperm viability, progressive motility and morphology, with increased total antioxidants levels in seminal plasma, suggesting a possible link between dietary antioxidant supplementation and sperm oxidative status.

Considering the detrimental consequences that elevated PUFAs levels might have on semen, Freitas and collaborators (Freitas, Bouéres, et al., 2016; Freitas, Pignataro, et al., 2016) investigated the effect of sixty‐day combined oral supplementation with antioxidants (vitamin E, selenium, L‐carnitine) and fatty acids (omega‐3 and omega‐6) on equine frozen semen (2 groups of 4 stallions each) in a cross‐over trial (60 days washout). Higher statistically significant values of spermatozoa progressive motility and integrity of plasma and acrosomal membranes were observed in supplemented group, as possible synergistic effect of antioxidant, L‐carnitine and selenium on spermatozoa kinetics. Likewise, the increase in plasma and acrosomal membrane integrity may be due to the combined effect of higher concentrations of PUFAs and the prevention of excessive lipid peroxidation by antioxidants (Freitas, Bouéres, et al., 2016; Freitas, Pignataro, et al., 2016).

## 4 BOTANICAL EXTRACTS

Medicinal plants have been used in traditional medicine since ancient times for the management of different diseases including reproductive disorders (Williams & Lamprecht, 2008). Nutraceuticals including herbs, fruits, vegetables and vitamins have been sponsored to ameliorate several aspects of male fertility, including sperm quality, erectile function, and libido (Crimmel et al., 2001; Ko & Sabanegh, 2014).

Among phytotherapy products, Maca (*Lepidium meyenii*), obtained by the root of a Brassicaceae plant native of Peru, is known since ancient time by Andean population for its nutritional and therapeutic properties (Wang & Zhu, 2019). In fact, besides being a medicament for osteoporosis, depression, anxiety, Maca exerts a beneficial effect on female reproduction, male sexual functions and spermatogenesis on different species of mammals (Da Silva Leitão Carvalho et al., 2020; Da Silva Leitão Peres et al., 2020; Tafuri, Cocchia, Vassetti, et al., 2019). The effect of dietary Lepidium meyenii on fresh and cooled stallion semen quality has been evaluated by Del Prete and collaborators (Del Prete et al., 2018). In the study, stallions' diet was supplemented with 20 g of Maca powder daily for a total of 60 days, with favourable effect on spermatozoa concentration, total count, and motility, and acrosome integrity. Furthermore, during cooling storage the decrease of motility and acrosome integrity was slower in the experimental group compared to controls (Del Prete et al., 2018). Differently from other authors who found a decrease in ROS and an increase in the antioxidant protection system following Maca supplementation, Del Prete and colleagues found no effect on oxidative status. In a different study, semen samples from the same stallions exhibited an improvement in sperm parameters including gel‐free volume, spermatozoa concentration, total and progressive motility, and acrosome integrity following Maca supplementation (Tafuri, Cocchia, Carotenuto, et al., 2019).

Van Dorland and collaborators added a commercial plasmolysed yeast product enriched with herbs, malt, honey and orange syrup to ten stallions' diet, other ten stallions served as controls (van Dorland et al., 2019). Sperm concentration, motility and velocity were assessed immediately, 24 and 48 hr after cooled storage. Membrane lipid peroxidation was evaluated after 24 and 48 hr of cooled storage, as well. The authors found that herbal supplementation did not statistically affect semen parameters and the antioxidant status of semen was only temporarily improved.

In recent years, algae have been found to be able to generate and accumulate omega‐3 long chain polyunsaturated fatty acids, representing a new source of PUFAs (Turon, 2013; Warren, 2015). However, up‐to‐date, only one study described the supplementation with heterotrophically grown microalgae enriched with Vitamin E and selenium in horses, highlighting an improvement of total and progressive motility in both fresh and cooled semen (Goedde, 2016).

## 5 DISCUSSION

The equine species is characterized by peculiar features of sperm biology and several attempts have been made to improve male fertility over years (Griffin et al., 2019). Starting from the last decade, a frame of research has been directed toward the use of nutritional supplements to improve the ability of equine semen to cope with cryodamage and oxidative stress (Hussain et al., 2018).

Based on the current literature, several strategies and nutraceutical compounds have been explored to provide beneficial effects on sperm quality.

The advantage of PUFAs as unique dietary supplement is fairly conflicting, minor improvements have been reported in some studies, whereas no improvement has been observed by other authors. These misleading results might be related to redox imbalance following excessive dietary fatty acids intake, which has been recognized as a major cause of male infertility (Peña et al., 2019; Sanocka & Kurpisz, 2004). According to the aforementioned studies, providing optimal levels of antioxidants is essential for the maintenance of normal reproductive functions, with the most promising results have been obtained in those studies investigating the combination of PUFAs and antioxidants.

In recent years, the use of plant‐derived nutraceuticals is gaining popularity in human and veterinary reproductive medicine (Garolla et al., 2020). Beyond *Lepidium meyenii*, yeast extracts, and algae there are a number of nutraceuticals that need to be explored, to ascertain their effects on reproductive function.

Investigating the benefits of nutraceuticals in equine reproduction represents a challenge which deserves to be attended. For those more adventurous which are considering the exploration of herbal extracts, it has to be clear that the number of active constituents can be influenced by several environmental factors, as well as by the plant parts and the subspecies used, paying attention to pollutants eventually present as pesticides, herbicides, and polychlorinated biphenyls (PCBs) residue which can have deleterious effect on gamete function (Harman, 2002).

## CONFLICT OF INTEREST

None of the authors have any conflict of interest to declare.

## AUTHOR CONTRIBUTIONS

Dr Bazzano developed the subject and wrote the manuscript, Prof. Laus contributed to write a section of the review, Prof. Spaterna contributed to write a section of the review, Dr Marchegiani supervised and completed the review.
